# *catena*-Poly[cobalt(II)-bis­(μ-2-amino­ethane­sulfonato)-κ^3^ *N*,*O*:*O*′;κ^3^ *O*:*N*,*O*′]

**DOI:** 10.1107/S1600536810038481

**Published:** 2010-09-30

**Authors:** Feng Yang, Xu-Hui Liu, Cheng-Qiang Zhao

**Affiliations:** a Key Laboratory for the Chemistry and Molecular Engineering of Medicinal Resources (Ministry Education of China), School of Chemistry & Chemical Engineering, Guangxi Normal University, Guilin 541004, People’s Republic of China; b Department of Chemistry and Life Science, Hechi University, Yizhou, Guangxi 546300, People’s Republic of People’s Republic of China

## Abstract

The hydro­thermally prepared title compound, [Co(C_2_H_6_NO_3_S)_2_]_*n*_, is isotypic with its Ni^II^ analogue. The Co^II^ cation is in a distorted octa­hedral environment, coordinated by four sulfonate O atoms and two N atoms from the taurine ligands. In comparison with the Ni^II^ analogue, the Co—N and Co—O bonds are longer than the Ni—N and Ni—O bonds, whereas all other bond lengths and angles as well as the hydrogen-bonding motifs are very similar in the two structures. The sulfonate groups doubly bridge symmetry-related Co^II^ atoms, forming polymeric chains along the *a* axis. N—H⋯O hydrogen bonding interactions consolidate the crystal packing.

## Related literature

For the isotypic Ni^II^ structure, see: Yang *et al.* (2010 ▶). For general background to taurine complexes and their derivatives, see: Bottari & Festa (1998 ▶); Zhang & Jiang (2002 ▶); Zhong *et al.* (2003 ▶); Cai *et al.* (2004 ▶); Jiang *et al.* (2005 ▶); Cai *et al.* (2006 ▶).
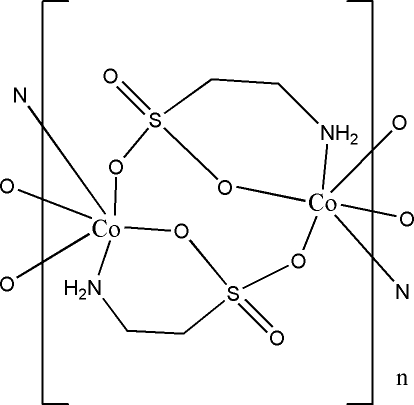


## Experimental

### 

#### Crystal data

[Co(C_2_H_6_NO_3_S)_2_]
*M* *_r_* = 307.21
Monoclinic, 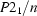

*a* = 5.139 (2) Å
*b* = 8.278 (4) Å
*c* = 11.737 (5) Å
β = 97.542 (6)°
*V* = 495.0 (4) Å^3^
*Z* = 2
Mo *K*α radiation
μ = 2.17 mm^−1^
*T* = 293 K
0.45 × 0.25 × 0.10 mm

#### Data collection

Bruker SMART APEX CCD area-detector diffractometer
Absorption correction: multi-scan (*SADABS*; Bruker, 1999 ▶) *T*_min_ = 0.527, *T*_max_ = 0.805
2173 measured reflections
974 independent reflections
931 reflections with *I* > 2σ(*I*)
*R*_int_ = 0.032

#### Refinement

*R*[*F* ^2^ > 2σ(*F* ^2^)] = 0.031
*wR*(*F* ^2^) = 0.083
*S* = 1.11
974 reflections
77 parameters
H atoms treated by a mixture of independent and constrained refinement
Δρ_max_ = 0.61 e Å^−3^
Δρ_min_ = −0.74 e Å^−3^

### 

Data collection: *SMART* (Bruker, 1999 ▶); cell refinement: *SAINT* (Bruker, 1999 ▶); data reduction: *SAINT*; program(s) used to solve structure: *SHELXS97* (Sheldrick, 2008 ▶); program(s) used to refine structure: *SHELXL97* (Sheldrick, 2008 ▶); molecular graphics: *SHELXTL* (Sheldrick, 2008 ▶); software used to prepare material for publication: *SHELXTL*.

## Supplementary Material

Crystal structure: contains datablocks I, global. DOI: 10.1107/S1600536810038481/zq2061sup1.cif

Structure factors: contains datablocks I. DOI: 10.1107/S1600536810038481/zq2061Isup2.hkl

Additional supplementary materials:  crystallographic information; 3D view; checkCIF report

## Figures and Tables

**Table 1:** Selected bond lengths (Å)

Co1—N1^i^	2.112 (2)
Co1—N1^ii^	2.112 (2)
Co1—O1^i^	2.1231 (18)
Co1—O1^ii^	2.1231 (18)
Co1—O2	2.1473 (18)
Co1—O2^iii^	2.1473 (18)

Symmetry codes: (i) 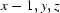; (ii) 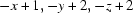; (iii) 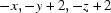.

**Table 2:** Hydrogen-bond geometry (Å, °)

*D*—H⋯*A*	*D*—H	H⋯*A*	*D*⋯*A*	*D*—H⋯*A*
N1—H1*C*⋯O3^iv^	0.86 (3)	2.43 (3)	3.148 (3)	142 (3)
N1—H1*D*⋯O3^v^	0.86 (3)	2.35 (3)	3.135 (3)	151 (3)

Symmetry codes: (iv) 
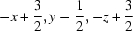
; (v) 
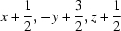
.

**Fig. 1.**
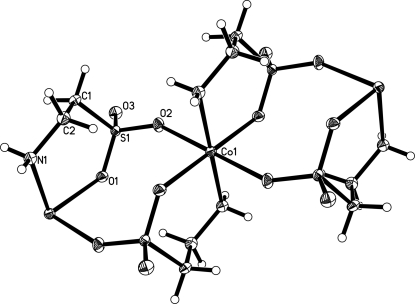
A segment of the polymeric structure of (I) with 30% probability displacement ellipsoids (arbitrary spheres for H atoms)

**Fig. 2.**
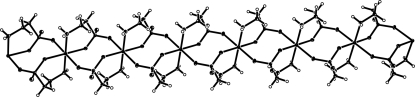
The one-dimensional polymeric chain of the title complex

## supplementary crystallographic information

### Comment

Taurine, an amino acid containing sulfur, is indispensable to human beings because of its important physiological functions (Bottari & Festa, 1998). Some metal complexes of the deprotonated sulfonic acid-type amino-acid taurine, C_2_H_6_NO_3_S^-^, have been reported (Cai *et al.,* 2004; Jiang *et al.,* 2005; Cai *et al.*, 2006). As part of our investigations into novel structures of taurine complex, we have synthesized the title compound, a new Co^II^ complex.

The coordinated modes of the title compound are similar to our previously reported Ni^II^ structure (Yang *et al.,* 2010). As shown in Fig. 1, the Co^II^ atom is coordinated by four sulfonate O atoms and to two N atoms of the taurine ligands, displaying a distorted octahedral coordination geometry. Neighbouring Co^II^ atoms are bridged by two sulfonate anions to form zigzag polymeric chains along the *a* axis, as shown in Fig. 2. The polymeric chain has a repeat unit formed by two taurine ligands and two Co^II^ atoms related by an inversion centre, which coincides with the centre of the eight-membered Co_2_S_2_O_4_ ring. The shortest distance between two Co atoms is 5.139 (6) Å.

In the structure of the title compound there are two symmetry-independent 'active' H atoms; both of them belong to the NH_2_ group of the taurine ligand. They form intramolecular hydrogen bonds with sulfonate atom O_3_.

### Experimental

A solution of taurine (1.0 mmol) and KOH (1.0 mmol) in anhydrous methanol (10 ml) was added slowly to a solution of Co(CH_3_COO)_2_ (1.0 mmol) in anhydrous methanol (10 ml). After stirring for 10 min, it was then dropped into a 25 ml Teflon-lined stainless steel reactor and heated at 383 K for six days. Thereafter, the reactor was slowly cooled to room temperature and pink block-shaped crystals suitable for X-ray diffraction were collected.

### Refinement

The H atoms bound to C atoms were positioned geometrically with C—H = 0.97 Å and included in the refinement in the riding-model approximation with *U*_iso_(H) = 1.2*U*_eq_(C). The H atoms bound to N were located in a difference Fourier map and freely refined  with *U*_iso_(H) = 1.2*U*_eq_(N).

### Crystal data

**Table:**

[Co(C_2_H_6_NO_3_S)_2_]	*F*(000) = 314
*M**_r_* = 307.21	*D*_x_ = 2.061 Mg m^−^^3^
Monoclinic, *P*2_1_/*n*	Mo *K*α radiation, λ = 0.71073 Å
Hall symbol: -P 2yn	Cell parameters from 717 reflections
*a* = 5.139 (2) Å	θ = 2.5–27.6°
*b* = 8.278 (4) Å	µ = 2.17 mm^−^^1^
*c* = 11.737 (5) Å	*T* = 293 K
β = 97.542 (6)°	Prism, red
*V* = 495.0 (4) Å^3^	0.45 × 0.25 × 0.10 mm
*Z* = 2	

### Data collection

**Table:**

Bruker SMART APEX CCD area-detector diffractometer	974 independent reflections
Radiation source: fine-focus sealed tube	931 reflections with *I* > 2σ(*I*)
graphite	*R*_int_ = 0.032
φ and ω scans	θ_max_ = 26.0°, θ_min_ = 3.0°
Absorption correction: multi-scan (*SADABS*; Bruker, 1999)	*h* = −5→6
*T*_min_ = 0.527, *T*_max_ = 0.805	*k* = −10→10
2173 measured reflections	*l* = −14→11

### Refinement

**Table:**

Refinement on *F*^2^	Secondary atom site location: difference Fourier map
Least-squares matrix: full	Hydrogen site location: inferred from neighbouring sites
*R*[*F*^2^ > 2σ(*F*^2^)] = 0.031	H atoms treated by a mixture of independent and constrained refinement
*wR*(*F*^2^) = 0.083	*w* = 1/[σ^2^(*F*_o_^2^) + (0.0517*P*)^2^ + 0.269*P*] where *P* = (*F*_o_^2^ + 2*F*_c_^2^)/3
*S* = 1.11	(Δ/σ)_max_ = 0.004
974 reflections	Δρ_max_ = 0.61 e Å^−^^3^
77 parameters	Δρ_min_ = −0.74 e Å^−^^3^
0 restraints	Extinction correction: *SHELXL97* (Sheldrick, 2008), Fc^*^=kFc[1+0.001xFc^2^λ^3^/sin(2θ)]^-1/4^
Primary atom site location: structure-invariant direct methods	Extinction coefficient: 0.060 (5)

### Special details

**Table:**

Geometry. All e.s.d.'s (except the e.s.d. in the dihedral angle between two l.s. planes) are estimated using the full covariance matrix. The cell e.s.d.'s are taken into account individually in the estimation of e.s.d.'s in distances, angles and torsion angles; correlations between e.s.d.'s in cell parameters are only used when they are defined by crystal symmetry. An approximate (isotropic) treatment of cell e.s.d.'s is used for estimating e.s.d.'s involving l.s. planes.
Refinement. Refinement of *F*^2^ against ALL reflections. The weighted *R*-factor *wR* and goodness of fit *S* are based on *F*^2^, conventional *R*-factors *R* are based on *F*, with *F* set to zero for negative *F*^2^. The threshold expression of *F*^2^ > σ(*F*^2^) is used only for calculating *R*-factors(gt) *etc*. and is not relevant to the choice of reflections for refinement. *R*-factors based on *F*^2^ are statistically about twice as large as those based on *F*, and *R*- factors based on ALL data will be even larger.

### Fractional atomic coordinates and isotropic or equivalent isotropic displacement parameters (Å^2^)

**Table:**

	*x*	*y*	*z*	*U*_iso_*/*U*_eq_	
Co1	0.0000	1.0000	1.0000	0.0180 (2)	
S1	0.46596 (10)	0.95782 (7)	0.81328 (4)	0.0169 (2)	
O1	0.6587 (3)	1.0572 (2)	0.88479 (14)	0.0225 (4)	
O2	0.2126 (3)	0.9583 (3)	0.85700 (15)	0.0258 (4)	
O3	0.4389 (4)	1.0020 (2)	0.69293 (16)	0.0270 (5)	
C1	0.5838 (5)	0.7567 (3)	0.82176 (19)	0.0235 (5)	
H1A	0.4506	0.6868	0.7816	0.028*	
H1B	0.7377	0.7502	0.7822	0.028*	
C2	0.6547 (4)	0.6942 (3)	0.9429 (2)	0.0239 (5)	
H2A	0.5260	0.7317	0.9904	0.029*	
H2B	0.6508	0.5771	0.9423	0.029*	
N1	0.9179 (4)	0.7500 (3)	0.99249 (18)	0.0209 (4)	
H1C	1.028 (6)	0.708 (4)	0.952 (3)	0.025*	
H1D	0.958 (5)	0.710 (4)	1.060 (3)	0.025*	

### Atomic displacement parameters (Å^2^)

**Table:**

	*U*^11^	*U*^22^	*U*^33^	*U*^12^	*U*^13^	*U*^23^
Co1	0.0176 (3)	0.0184 (3)	0.0184 (3)	−0.00135 (15)	0.00344 (18)	−0.00035 (15)
S1	0.0165 (3)	0.0198 (3)	0.0151 (3)	0.0000 (2)	0.0049 (2)	−0.0009 (2)
O1	0.0242 (8)	0.0185 (8)	0.0244 (8)	−0.0001 (7)	0.0018 (6)	−0.0018 (7)
O2	0.0201 (9)	0.0356 (10)	0.0236 (9)	0.0009 (7)	0.0094 (7)	0.0004 (7)
O3	0.0310 (10)	0.0332 (11)	0.0176 (9)	0.0002 (7)	0.0060 (7)	0.0020 (6)
C1	0.0257 (12)	0.0195 (11)	0.0251 (12)	0.0017 (9)	0.0029 (9)	−0.0067 (9)
C2	0.0247 (12)	0.0182 (11)	0.0299 (12)	−0.0026 (9)	0.0083 (9)	0.0021 (9)
N1	0.0229 (10)	0.0208 (10)	0.0195 (9)	−0.0010 (9)	0.0042 (7)	0.0016 (8)

### Geometric parameters (Å, °)

**Table:**

Co1—N1^i^	2.112 (2)	O1—Co1^iv^	2.1231 (18)
Co1—N1^ii^	2.112 (2)	C1—C2	1.512 (3)
Co1—O1^i^	2.1231 (18)	C1—H1A	0.9700
Co1—O1^ii^	2.1231 (18)	C1—H1B	0.9700
Co1—O2	2.1473 (18)	C2—N1	1.475 (3)
Co1—O2^iii^	2.1473 (18)	C2—H2A	0.9700
S1—O3	1.4481 (19)	C2—H2B	0.9700
S1—O2	1.4610 (17)	N1—Co1^iv^	2.112 (2)
S1—O1	1.4642 (18)	N1—H1C	0.86 (3)
S1—C1	1.769 (3)	N1—H1D	0.86 (3)
			
N1^i^—Co1—N1^ii^	180.000 (1)	S1—O1—Co1^iv^	132.83 (11)
N1^i^—Co1—O1^i^	92.76 (7)	S1—O2—Co1	147.49 (11)
N1^ii^—Co1—O1^i^	87.24 (7)	C2—C1—S1	114.40 (16)
N1^i^—Co1—O1^ii^	87.24 (7)	C2—C1—H1A	108.7
N1^ii^—Co1—O1^ii^	92.76 (7)	S1—C1—H1A	108.7
O1^i^—Co1—O1^ii^	180.000 (1)	C2—C1—H1B	108.7
N1^i^—Co1—O2	85.93 (8)	S1—C1—H1B	108.7
N1^ii^—Co1—O2	94.07 (8)	H1A—C1—H1B	107.6
O1^i^—Co1—O2	90.03 (7)	N1—C2—C1	111.05 (18)
O1^ii^—Co1—O2	89.97 (7)	N1—C2—H2A	109.4
N1^i^—Co1—O2^iii^	94.07 (8)	C1—C2—H2A	109.4
N1^ii^—Co1—O2^iii^	85.93 (8)	N1—C2—H2B	109.4
O1^i^—Co1—O2^iii^	89.97 (7)	C1—C2—H2B	109.4
O1^ii^—Co1—O2^iii^	90.03 (7)	H2A—C2—H2B	108.0
O2—Co1—O2^iii^	180.000 (1)	C2—N1—Co1^iv^	119.40 (15)
O3—S1—O2	111.46 (11)	C2—N1—H1C	107 (2)
O3—S1—O1	112.86 (11)	Co1^iv^—N1—H1C	106 (2)
O2—S1—O1	111.35 (11)	C2—N1—H1D	109.7 (19)
O3—S1—C1	106.30 (10)	Co1^iv^—N1—H1D	109 (2)
O2—S1—C1	107.27 (12)	H1C—N1—H1D	105 (3)
O1—S1—C1	107.20 (11)		

Symmetry codes: (i) *x*−1, *y*, *z*; (ii) −*x*+1, −*y*+2, −*z*+2; (iii) −*x*, −*y*+2, −*z*+2; (iv) *x*+1, *y*, *z*.

### Hydrogen-bond geometry (Å, °)

**Table:**

*D*—H···*A*	*D*—H	H···*A*	*D*···*A*	*D*—H···*A*
N1—H1C···O3^v^	0.86 (3)	2.43 (3)	3.148 (3)	142 (3)
N1—H1D···O3^vi^	0.86 (3)	2.35 (3)	3.135 (3)	151 (3)

Symmetry codes: (v) −*x*+3/2, *y*−1/2, −*z*+3/2; (vi) *x*+1/2, −*y*+3/2, *z*+1/2.
